# Identifying Risk Factors and Patterns for Early Recurrence of Pancreatic Neuroendocrine Tumors: A Multi-Institutional Study

**DOI:** 10.3390/cancers13092242

**Published:** 2021-05-07

**Authors:** Charlotte M. Heidsma, Diamantis I. Tsilimigras, Flavio Rocha, Daniel E. Abbott, Ryan Fields, George A. Poultsides, Clifford S. Cho, Alexandra G. Lopez-Aguiar, Zaheer Kanji, Alexander V. Fisher, Bradley A. Krasnick, Kamran Idrees, Eleftherios Makris, Megan Beems, Casper H. J. van Eijck, Elisabeth J. M. Nieveen van Dijkum, Shishir K. Maithel, Timothy M. Pawlik

**Affiliations:** 1 Department of Surgery, The Ohio State University Wexner Medical Center, 410 W 10th Ave, Columbus, OH 43210, USA; c.m.heidsma@amsterdamumc.nl (C.M.H.); diamantis.tsilimigras@osumc.edu (D.I.T.); 2 Department of Surgery, Amsterdam University Medical Centers, University of Amsterdam, Meibergdreef 9, 1105AZ Amsterdam, The Netherlands; e.j.nieveenvandijkum@amsterdamumc.nl; 3 Department of Surgery, Virginia Mason Medical Center, 1100 9th Ave, Seattle, WA 98101, USA; flavio.rocha@virginiamason.org (F.R.); Zaheer.Kanji@virginiamason.org (Z.K.); 4 Department of Surgery, University of Wisconsin School of Medicine and Public Health, 750 Highland Ave, Madison, WI 53726, USA; abbot@surgery.wisc.edu (D.E.A.); afisher2@uwhealth.org (A.V.F.); 5 Department of Surgery, Washington University School of Medicine, 660 S Euclid Ave, St. Louis, MO 63110, USA; rcfields@wustl.edu (R.F.); krasnickb@wudosis.wustl.edu (B.A.K.); 6 Department of Surgery, Stanford University, 300 Pasteur Drive, Stanford, CA 94305-2200, USA; George.Poultsides@stanford.edu (G.A.P.); emakris@stanford.edu (E.M.); 7 Division of Hepatopancreatobiliary and Advanced Gastrointestinal Surgery, Department of Surgery, University of Michigan, 1500 E. Medical Center Drive, Ann Arbor, MI 48109, USA; cliffcho@med.umich.edu (C.S.C.); mbeems@umich.edu (M.B.); 8 Division of Surgical Oncology, Department of Surgery, Winship Cancer Institute, Emory University, 1365 Clifton Rd, Atlanta, GA 30322, USA; alexandra.grace.nicole.lopez-aguiar@emory.edu (A.G.L.-A.); smaithe@emory.edu (S.K.M.); 9 Division of Surgical Oncology, Department of Surgery, Vanderbilt University, 1211 Medical Center Drive, Nashville, TN 37232, USA; kamran.idrees@vanderbilt.edu; 10 Department of Surgery, Erasmus Medical Center, Doctor Molewaterplein 40, 3015 GD Rotterdam, The Netherlands; c.vaneijck@erasmusmc.nl

**Keywords:** pancreatic neuroendocrine tumor, recurrence, risk-factors

## Abstract

**Simple Summary:**

Approximately 30% of patients with a pancreatic neuroendocrine tumor (pNET) will develop metastases. Curative-intent treatment largely involves resection. Identifying patients with early recurrence (ER) following resection might help tailor adjuvant therapies and the surveillance intensity. The aim of this retrospective study was to determine an evidence-based cut-off value for ER, and to explore risk factors associated with ER. ER was identified 18 months after surgery. Tumor size (OR 1.20, 95% CI 1.05–1.37, *p* = 0.007) and positive lymph nodes (OR 4.69, 95%CI 1.41–15.58, *p* = 0.01) were independently associated with ER. Patients with ER had lower post-recurrence free survival and overall survival than patients with late recurrence. These data support intensive follow-up shortly after surgery, and adjuvant therapy may help improve survival in pNET patients with ER after surgery.

**Abstract:**

Background: Identifying patients at risk for early recurrence (ER) following resection for pancreatic neuroendocrine tumors (pNETs) might help to tailor adjuvant therapies and surveillance intensity in the post-operative setting. Methods: Patients undergoing surgical resection for pNETs between 1998–2018 were identified using a multi-institutional database. Using a minimum *p*-value approach, optimal cut-off value of recurrence-free survival (RFS) was determined based on the difference in post-recurrence survival (PRS). Risk factors for early recurrence were identified. Results: Among 807 patients who underwent curative-intent resection for pNETs, the optimal length of RFS to define ER was identified at 18 months (lowest *p*-value of 0.019). Median RFS was 11.0 months (95% 8.5–12.60) among ER patients (*n* = 49) versus 41.0 months (95% CI: 35.0–45.9) among non-ER patients (*n* = 77). Median PRS was worse among ER patients compared with non-ER patients (42.6 months vs. 81.5 months, *p* = 0.04). On multivariable analysis, tumor size (OR: 1.20, 95% CI: 1.05–1.37, *p* = 0.007) and positive lymph nodes (OR: 4.69, 95% CI: 1.41–15.58, *p* = 0.01) were independently associated with ER. Conclusion: An evidence-based cut-off value for ER after surgery for pNET was defined at 18 months. These data emphasized the importance of close follow-up in the first two years after surgery.

## 1. Introduction

Although rare, the incidence of pancreatic neuroendocrine tumors (pNETs) in the United States has increased over the last decade [1]. PNETs are characterized by heterogeneous behavior as some tumors can be associated with wide-spread metastatic disease, while other pNETs can remain indolent for decades [1,2]. Curative-intent treatment of pNETs largely involves resection [3,4]. In turn, the incidence of recurrence following resection of pNET has been reported to be as high as 20–30% [5,6]. Certain histological and morphological features have been associated with risk of recurrence following curative resection [7,8,9,10]. Among patients who have experienced a recurrence, the prognosis of patients is generally worse. Data on patterns of recurrence, as well as timing of recurrence, following resection of pNETs have not, however, been previously well characterized [4,5,6,11,12].

The timing of recurrence may be a particularly important factor relative to long-term survival. Specifically, patients with other types of hepatopancreatobiliary (HPB) tumors such as hepatocellular carcinoma, intrahepatic cholangiocarcinoma, and gallbladder cancer who recurred within 12–24 months of resection had a very poor overall survival [13,14,15]. To date, the impact of early recurrence (ER) after curative-intent resection for pNETs has not been well investigated. Furthermore, classification of patients with ER has not been the topic of investigation among patients with pNETS. In fact, data on the optimal cut-off period to define ER versus late recurrence (non-ER), as well as possible predictors of ER, among patients undergoing resection of pNETs have not been reported. These data may be important to identify a subset of patients prone to ER and early metastasis soon after surgery who may benefit from primary tumor resection (with potential associated morbidity and mortality), neoadjuvant treatment, or a more intensive follow-up regimen.

Systemic treatment options for patients with pNETs has improved over the last decade. In particular, options for systemic treatment now include somatostatin-analogues (SSA), Peptide Receptor Radionuclide Therapy (PRRT), or chemotherapy. In fact, neoadjuvant systemic chemotherapy alone or in combination with PRRT have demonstrated favorable outcomes [16,17,18]. In particular, chemotherapy, which is more widely available and applicable to a larger subset of patients, may be of value in patients with high risk pNETs. Other investigators have also suggested that patients at high risk of recurrence after curative-intent resection of pNETs should be considered for adjuvant therapy [19]. To date, there is no consensus about which patients may benefit from (neo)adjuvant therapy following resection of pNETs. Therefore, determining risk factors for ER and non-ER may help distinguish which patients may benefit from alternative up-front treatment strategies, as well as possible adjuvant therapy and closer or longer postoperative surveillance. The objective of the current study was to characterize factors associated with the risk for ER versus non-ER following curative-intent resection of pNETs, as well as define overall patterns of recurrence using a large, multi-institutional database.

## 2. Materials and Methods

### 2.1. Study Cohort and Data Collection

Patients undergoing surgical resection for PNETs between 1998 and 2018 were identified using a multi-institutional database. Data were obtained on patients who underwent surgery in one of eight tertiary institutions comprising the United States Neuroendocrine Tumor Study Group (US-NETSG) (The Ohio State University Wexner Medical Center, Columbus, OH, USA; Virginia Mason Medical Center, Seattle, WA, USA; Washington University School of Medicine, St Louis, MO, USA; University of Wisconsin School of Medicine and Public Health, Madison, WI, USA; Vanderbilt University, Nashville, TN, USA; Stanford University, Stanford, CA, USA; University of Michigan, Ann Arbor, MI, USA; Winship Cancer Institute, Emory University, Atlanta, GA, USA) and two tertiary centers in the Netherlands (Erasmus Medical Center, Rotterdam, The Netherlands; Academic Medical Center, Amsterdam University Medical Centers, Amsterdam, The Netherlands). Prior to surgery, all patients had undergone an abdominal computed tomography (CT) scan or abdominal magnetic resonance imaging (MRI) scan. An abdominal MRI was generally performed in the event that the patient was not eligible for abdominal CT (e.g., young age), or to clarify indeterminate findings. Prior to 2015, somatostatin receptor imaging (SRS) was not routinely used in the diagnostic workup of pNET patients. In later time periods, SRS imaging was, however, performed as an adjunct to CT or MRI.

All patients were diagnosed with a pNET by final histologic examination. The databases (US NETSG and Dutch) were reviewed and updated definitions (e.g., International Study Group on Pancreatic Surgery definitions [20]) were applied to all data; pathological specimens were re-evaluated by an experienced local pathologist. Patients with a grade 3 tumor, genetic syndrome, an R2 resection or metastases at time of diagnosis, as well as patients with missing data on recurrence status were excluded. Furthermore, patients with a recurrence within 3 months after surgery were excluded to mitigate the chance of undetected synchronous metastases at the time of surgery, or a grossly incomplete resection. The Institutional Review Boards of each participating institution approved the study.

A functional tumor was defined as a lesion associated with symptoms related to hormone overproduction, including insulinoma, glucagonoma, gastrinoma, VIPoma, and somatostatinoma [21]. An R0 resection was defined as a minimum margin width of >1 mm; an R1 resection was defined as the microscopic presence of tumor at the margin or a minimum margin length of ≤1 mm [22]. Grade 1 tumors had a Ki-67 index of <3%, grade 2 tumors had a Ki-67 index of 3–20%. Pathologic tumor T and N categories were defined according to the American Joint Committee on Cancer (AJCC) 8th edition manual [23]. Severe post-operative complications were defined as Clavien-Dindo grade ≥3 within 90-days after surgery [24]. Recurrence-free survival (RFS) was defined as the time duration from the date of initial surgery to tumor recurrence. Post-recurrence survival (PRS) was defined as the time from recurrence until last follow-up or death. Overall survival (OS) after recurrence was defined as the time duration from the date of recurrence after surgery to patient death or the end of follow-up.

### 2.2. Follow Up and Pattern of Recurrence

All patients were followed regularly at each participating institution. The follow-up protocol at each center was once every 3–6 months within the first 3 years after the operation and then once every 6 months until at least year five, after which screening occurred annually depending on the participating center. Follow-up imaging was typically performed using CT of the abdomen. Additional imaging (e.g., magnetic resonance imaging, endoscopic ultrasound, somatostatin receptor imaging (SRS) such as octreotide scintigraphy or 68-Gallium Dotatate PET scan) was performed for cases in which doubt about disease progression existed.

A recurrent pNET was defined as identification of metastatic imaging findings on postoperative surveillance (i.e., routine CT abdomen) or biopsy proven disease. The initial recurrence site was identified for purposes of classification. The initial recurrence sites were classified into three mutually exclusive patterns: local recurrence, distant recurrence, and local + distant recurrence. Local recurrence was defined as the initial recurrence only if the recurrence occurred in the remnant pancreas, at the cut surface or peri-pancreatic lymph nodes; distant (liver) recurrence was defined if the initial recurrence occurred only in the liver [12]. Recurrence in organs other than the pancreas, liver, and lymph nodes were reported in aggregate due to the low occurrence rates.

### 2.3. Statistical Analysis

Continuous variables were expressed as medians with interquartile ranges (IQR) or means and standard deviation (SD); categorical variables were expressed as totals and percentages. Statistical analyses were performed with the independent *t*-test, Mann–Whitney *U* test, *χ*^2^ test or Fisher exact test as appropriate. RFS and PRS after recurrence were estimated using the Kaplan–Meier method and compared by log-rank analysis. For the purpose of this study, a clinically relevant cut-off was determined based on differences in PRS among different RFS groups, as previously described by Groot et al. [25]. A minimum *p*-value approach was used to evaluate the optimal threshold of RFS to divide the patients into early versus late recurrence cohorts based on the length of PRS. Using this approach, the log-rank test was performed relative to different lengths of RFS to determine the optimal cut-off point with the lowest *p*-value. Associations between potential risk factors and early and late recurrence of pNET were assessed by univariable logistic regression. Variables with a *p*-value of <0.10 were included as a covariate in two separate multivariable logistic regression models. Results were presented as an odds ratio (OR) with corresponding 95% confidence interval (95% CI). A two-tailed *p*-value of <0.05 was considered statistically significant. Statistical analysis was performed using SPSS Version 22.0 (IBM Corporation, Armonk, NY, USA) and R version 3.4.3 (cran.r-project.org, accessed date: 10 February 2021). Statistical significance was assessed at α = 0.05 (two-tailed).

## 3. Results

### 3.1. Patient Cohort

Among 807 patients who underwent curative intent surgery for a pNET, median patient age was 58 years (IQR, 49–66) and roughly one-half was male (*n* = 406, 50.3%); a small subset of patients had a functional tumor (*n* = 90, 11.3%) (Table 1). The average tumor size was 2.2 cm (IQR 1.4–3.8) and the tumor location was distributed roughly equally among the head (*n* = 246, 30.5%), body (*n* = 238, 29.5%), and tail (*n* = 321, 39.8%) of the pancreas. Distal pancreatectomy was the most common procedure (*n* = 451, 59.3%) followed by classic or pylorus-preserving pancreaticoduodenectomy (*n* = 246, 32.3%); a subset of patients underwent a parenchyma preserving resection (i.e., enucleation) (*n* = 64, 8.4%). At the time of surgery, 719 (89.1%) patients underwent a lymphadenectomy with a median of nine nodes (IQR 4–15) examined. On final pathology, 177 (21.9%) patients had metastatic lymph nodes, whereas the majority of patients (*n* = 687, 85.1%) had an R0 margin status; roughly one-half of patients (*n* = 437, 54.2%) had a grade 1 tumor. In the post-operative period, 197 (24.4%) patients experienced a severe (Clavien-Dindo III–V) complication. Of note, the majority of patients underwent surgery between 2011–2016 (*n* = 416, 51.5%) followed by 2006–2010 (*n* = 278, 34.4%), and then 1998–2005 (*n* = 113, 14.0%).

### 3.2. Time to Recurrence

**Figure 1:**
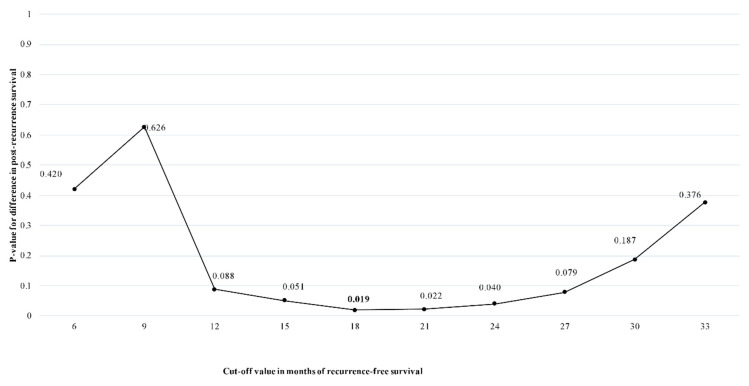
The cut-off value in months of RFS determined by difference in PRS for patients with a pNET.

**Figure 2:**
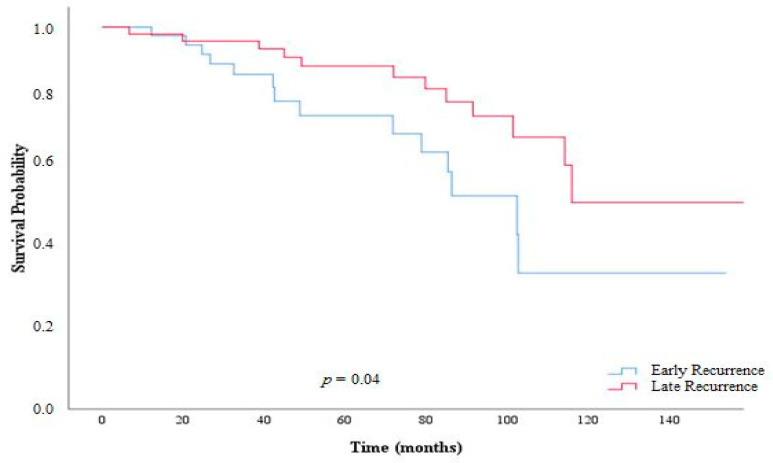
Kaplan–Meier curves demonstrating differences in PRS among patients with early versus late recurrence.

At the time of last follow-up, 127 patients (15.7%) had recurred with a median RFS of 26.0 months (95% CI 27.7–37.9). Median OS was 63.0 months (95% CI 49.0–81.5) among patients with no recurrence versus 37.7 months (95% CI 34.9–40.9) among patients who recurred. The minimum *p*-value analysis by log-rank test determined an optimal cut-off period of 18 months to differentiate patients with ER versus non-ER. Specifically, the *p*-value in the log-rank test was minimum at *p* = 0.019 to categorize 49 (6.1%) patients as potential ER (median RFS: 11.0 months, 95% CI 8.5–12.6) versus 77 (9.5%) potential non-ER (median RFS: 41 months, 95% CI 35.0–45.9) (Table 2, Figure 1). PRS of patients with ER was 10.2 months (95% CI 8.5–12.6) versus 43.4 months (95% CI 36.3–52.0) in non-ER patients. The *p*-values for each evaluated cut-off month are noted in Supplementary Table S1. Perhaps not surprisingly, patients with ER had a worse median PRS (42.6 months, 95% CI 37.2–61.0) versus non-ER (i.e., recurrence >18 months after surgery) (81.5 months, 95% CI 58.7–91.5) (*p* = 0.04) (Figure 2). The overall incidence of ER was largely comparable among patients in the years 1998–2005 (*n* = 12, 10.6%) versus 2006–2010 (*n* = 19, 6.8%) versus 2011–2016 (*n* = 18, 4.3%) (*p* = 0.04).

### 3.3. Risk Factors for Recurrence

**Figure 3:**
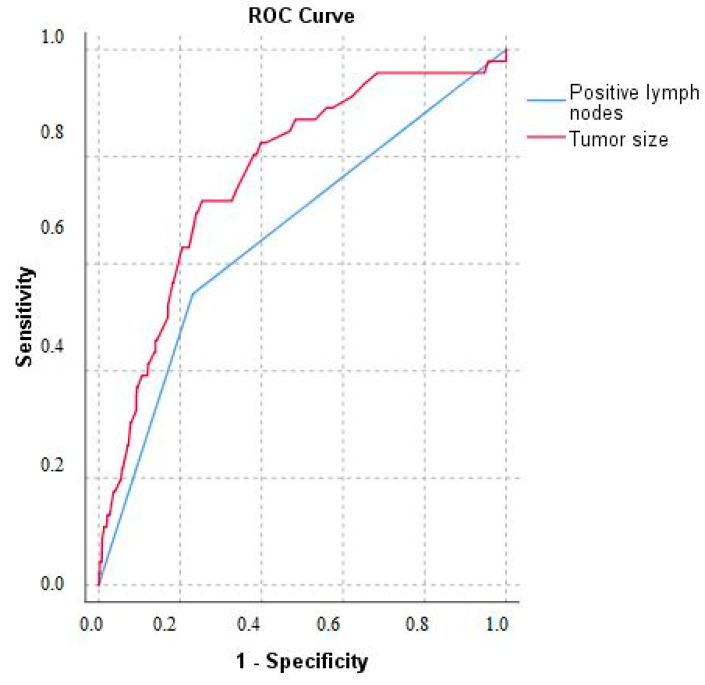
The receiver-operating curve of the risk factors lymph node invasion and tumor size on ER in patients with a pNET.

Characteristics of patients with and without ER are summarized in Table 2. Risk factors for recurrence are summarized in Table 3. Interestingly, on multivariable logistic regression analysis, tumor size (OR 1.17, 95% CI 1.05–1.30, *p =* 0.004), tumor grade (OR 2.82, 95% CI 1.38–5.79, *p =* 0.005), and metastatic lymph nodes (OR 2.32, 95% CI 1.02–5.25, *p* = 0.045) were independently associated with recurrence. In contrast, risk of ER within 18 months was associated with tumor size (OR 1.20, 95% CI 1.05–1.37, *p* = 0.007) and metastatic lymph node status (OR 4.69, 95% CI 1.41–15.58, *p* = 0.01). Risk of non-ER after 18 months was independently associated only with tumor grade (OR 2.55, 95% CI 1.03–6.34, *p =* 0.04). Tumor size was the strongest predictor for ER with an AUC of 0.766, while metastatic lymph nodes had an AUC of 0.656 (Figure 3). Among patients with a pNET > 2 cm, tumor size remained an independent risk factor (OR 1.17, 95% CI 1.01–1.36, *p* = 0.03) for ER, along with tumor grade (OR 3.34, 95% CI 1.02–11.00, *p* = 0.05) and metastatic lymph node disease (OR 4.84, 95% CI 1.36–17.26, *p* = 0.02) (Table S1).

### 3.4. Patterns of Recurrence

**Figure 4:**
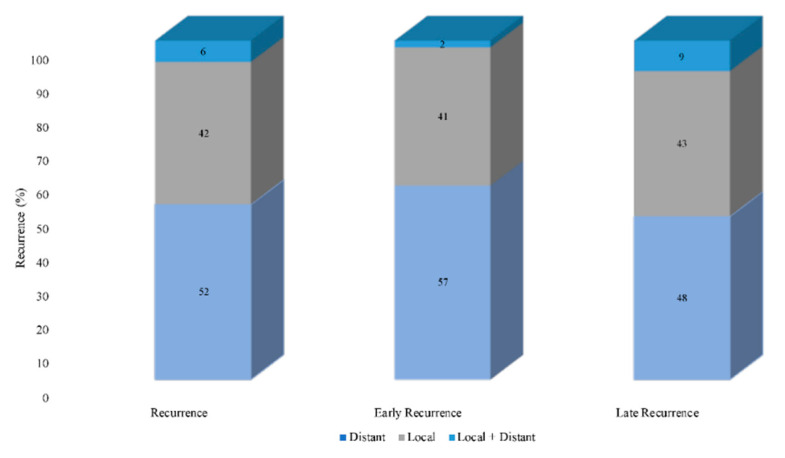
Distribution of the primary site of recurrence (local, distant, local + distant) in patients with a pNET, stratified into those with early versus late recurrence.

Among patients who recurred (*n* = 127), most individuals experienced distant recurrence (*n* = 66, 51.6%) followed by local recurrence (*n* = 53, 42.1%) or local + distant recurrence (*n* = 8, 6.3%). Median RFS was comparable among patients with local recurrence (29.0 months, 95% CI 19.8–38.2) distant recurrence (24.3 months, 95% CI 16.7–31.9), as well as individuals with both local + distant recurrence (25.6 months, 95% CI 16.3–35.0; *p* = 0.53). Median PRS was comparable among patients with isolated distant recurrence (33.0 months, 95% CI 26.3–39.7), isolated local recurrence (27.0 months, 95% CI 6.1–47.9), and local + distant recurrence (39.0 months, 95% CI 0–87.8; *p* = 0.67). In addition, OS was comparable among patients with local recurrence (54.8 months, 95% CI 29.4–80.2), distant recurrence (68.8 months, 95% CI 49.0–88.5), as well as local + distant recurrence (56.2 months, 95% 18.0–94.5; *p* = 0.41). Of note, patterns of recurrence were comparable among patients with early and late recurrence (Figure 4, *p* = 0.24).

## 4. Discussion

Neuroendocrine tumors of the pancreas can exhibit heterogeneous behavior, making accurate prediction of unfavorable outcomes challenging. The prognosis of patients with a pNET is significantly impacted by disease recurrence, yet there is no evidence-based cut-off value for ER [6,10]. Patients at high risk for ER may, however, warrant adjuvant therapy in order to improve recurrence-free and overall survival, while non-ER patients may require different long-term follow-up strategies. To the best of our knowledge, the current study was the first to define an optimal cut-off value for ER, which was identified as an RFS interval of 18 months. Of note, patients with metastatic lymph nodes were at markedly higher risk of experiencing ER, as were patients with larger tumor size. In contrast, tumor grade was more associated with the risk of developing non-ER. Interestingly, recurrence patterns among patients with ER and non-ER were largely comparable.

Due to variations in the malignant potential of a pNET, tumor metastases can occur from as early as one month after surgery up to several decades after resection [1,2,26]. Using differences in survival after recurrence, we sought to define a clinically relevant ER threshold to characterize patients at risk for short- versus long-term recurrence, as well as define overall survival among these different cohorts of patients. A cut-off value of 18 months was empirically defined as ER among patients who underwent resection of pNETs. Perhaps not surprisingly, OS was markedly lower among patients with ER compared with individuals with non-ER (42.5 months vs. 82.6 months, *p* < 0.01). PRS was also significantly lower among ER patients (10.2 months) compared with non-ER (43.4 months) patients. Tumor size and metastatic lymph node disease were independent risk factors for ER. Collectively, the data suggested that timing of recurrence impacted both OS and PRS.

Several studies have examined risk factors associated with recurrence among patients with a pNET [3,4,10,11,12,27]. Risk-factors for recurrence have included the presence of genetic syndrome, high tumor grade (grade 2 or 3), large tumor size, metastatic lymph nodes, lymphovascular invasion, and perineural invasion. However, these previous reports examined risk factors associated with recurrence at any time in the post-operative course—both early and late recurrence. In contrast, the current study specifically examined the risk factors associated with recurrence relative to the timing of the recurrence. Interestingly, certain factors such as the presence of metastatic lymph nodes was more associated with ER, while tumor grade impacted non-ER risk of recurrence. The presence of nodal metastasis in other malignancies such as breast or colorectal cancer has similarly been associated with ER [28,29]. Several previous models to predict recurrence after curative intent resection of pNETs have included lymph node metastasis, substantiating the strong prognostic power associated with this clinical factor [5,10,30]. In fact, a recent systematic review reported that lymph node metastases are prevalent even in G1 (15.8%) and small pNETs (11.5%) [31]. Yet, the importance of (extended) lymphadenectomy in pNETs is still debated since routine formal pancreatectomy with lymphadenectomy may represent overtreatment, particularly in small, G1 pNETs. In addition, extended lymphadenectomy has not been correlated with improved OS [32,33], and no universal threshold for the minimum number of nodes to be resected has been determined.

The majority of patients in the current cohort underwent lymphadenectomy (median nodes resected was nine), with only a small proportion undergoing parenchyma sparing surgery. Previously, Conrad et al. reported that extended lymph node dissection (i.e., >10 nodes) was not associated with better survival and that limited lymph node resection was favorable for select patients [32]. In turn, our own group reported a marked increase in the trend of lymph node dissection over time. In particular, extended lymph node dissection (i.e., >12 nodes) was more frequently performed among patients with tumors > 2 cm, located in the pancreatic head [33]. Similarly, Zhang et al. advocated for the examination of at least 11 lymph nodes to classify the N stage accurately in pNETs [34]. As such, the majority of patients in the current cohort may have had an “inadequate” lymph node dissection. It is important to note, however, that three different guidelines recommend various approaches to lymph node dissection. Specifically, the National Comprehensive Cancer Network (NCCN) guidelines [35] advise a lymphadenectomy for all pNET > 2 cm, the North American Neuroendocrine Tumor Society (NANETS) guidelines recommend lymph node dissection for all NF-PNET [36], while the European Neuroendocrine Tumor Society (ENETS) guidelines provide no clear recommendation on the topic [21]. As such, when considering lymph node dissection in pNETs, we support the general recommendation, based in part on findings in the current study, that lymphadenectomy should be performed at the time of formal pancreatectomy. For patients with a pNET eligible for pancreas persevering resection, lymph node sampling of suspicious nodes is advised. Future studies are needed to examine the necessity of lymphadenectomy among low-risk patients with a pNET—preferably with prospective randomized clinically controlled trials.

We also noted that a larger pNET size was associated with a higher risk of ER. PNET size has been identified as an important prognostic factor, with a pNET < 2 cm able to be managed conservatively [1,37]. It remains debatable whether to wait-and-see is the best option since long-term results are lacking. Long-term surveillance may represent a higher burden than surgical resection, especially among young patients with a tumor located far from the pancreatic head and duct (i.e., possibility of enucleation or spleen preserving distal pancreatectomy). In contrast, extensive resection including lymphadenectomy may pose unnecessary risks among patients with small tumors who may never develop metastases. In the current study, in the sensitivity analysis of patients with tumor size >2 cm, size remained an independent risk factor for ER along with metastatic lymph nodes. Furthermore, among patients with pNET > 2 cm, those patients with grade 2 tumors were three-fold more likely to develop ER versus patients with a grade 1 pNET. More recently, Dong et al. has reported that overall tumor burden can predict recurrence following curative-intent resection of non-functional pNETs [38]. The strong predictive value of lymph node metastasis (OR 4.84) and tumor grade 2 (OR 3.34) in pNETs > 2 cm, substantiate stronger guideline recommendations to perform a lymphadenectomy in high-risk patients [21,35]. Of note, the predictive ability of lymph nodes and tumor size was very good (both AUC > 0.65) in the current study.

The current study had several limitations that should be considered when interpreting the results. Although the use of multi-institutional data increased the generalizability of the results, there were likely variations in patient selection, surgical procedures, as well as postoperative surveillance. Importantly, in the past decade, recognition of pNETs has improved immensely through both awareness among physicians, and through improved imaging techniques. This fact could have influenced the results, especially the incidence of ER detection. Interestingly, ER actually occurred more frequently among patients who underwent surgery before 2005. While the reason for this is likely multifactorial, one possible explanation could be that these patients were more likely to have manifestation of “early recurrence” related to missed disease at the time of surgery. While the cut-off value for ER was identified using empiric well-established methods, the 18 months definition of ER will need to be validated in other external cohorts of patients with pNETs.

## 5. Conclusions

In conclusion, an evidence-based cut-off value for ER after surgery for pNET was defined at 18 months. These data emphasized the importance of close follow-up in the first two years after surgery. While guidelines remain debated, adjuvant treatment may be appropriate for patients at risk of ER following resection for pNETs [22]. Data from the current study not only defined the incidence of ER, but also may help to identify which patients are at risk for ER following pNET resection.

## Figures and Tables

**Table 1:** Patient and tumor characteristics.

Characteristics	All Patients (*n* = 807)	No Recurrence (*n* = 681)	Recurrence (*n* = 127)	*p*
Patient				
Male, %	406 (50.3)	344 (50.5)	62 (49.2)	0.85
Age, ± SD	58 (49–66)	59 (49–66)	58 (48–66)	0.72
BMI, ± SD	27 (24–32)	28 (25–32)	25 (22–29)	**<0.01**
ASA, %				0.15
I	44 (5.7)	32 (4.7)	12 (9.5)	
II	355 (45.7)	305 (44.8)	50 (39.7)	
III	361 (46.5)	304 (44.6)	57 (45.2)	
IV	17 (2.2)	15 (2.2)	2 (1.6)	
Tumor				
Functional tumor, %	90 (11.3)	81 (11.9)	9 (7.1)	0.13
Symptomatic, %	381 (53.8)	322 (47.3)	59 (46.8)	0.08
Tumor size, IQR, cm	2.2 (1.4–3.8)	1.9 (1.3–3.5)	4.0 (2.5–6.9)	**<0.01**
Tumor Location, %				**<0.01**
Head	246 (30.5)	189 (27.8)	57 (45.2)	
Body	238 (29.5)	210 (30.8)	28 (22.2)	
Tail	321 (39.8)	281 (41.3)	40 (31.7)	
Multiple tumors, %	15 (2.1)	15 (2.2)	0	0.24
Type of resection				**<0.01**
Pancreatoduodenectomy	246 (32.3)	188 (27.6)	58 (46.0)	
Distal pancreatectomy	451 (59.3)	397 (58.3)	54 (42.9)	
Enucleation/Central	64 (8.4)	56 (8.2)	8 (6.3)	
Major venous/arterial resection	35 (4.9)	22 (3.2)	13 (10.3)	**<0.01**
Complications CD grade ≥ 3	197 (24.4)	159 (23.3)	38 (30.2)	0.12
Pathological				
Tumor Grade, %				**<0.01**
G1	437 (54.2)	399 (58.6)	38 (30.2)	
G2	211 (26.1)	158 (23.2)	53 (42.1)	
LVI, %	170 (21.1)	126 (18.5)	44 (34.9)	**<0.01**
PNI, %	124 (15.4)	98 (14.4)	26 (20.6)	**<0.01**
Resection Margin, %				**0.005**
R0	687 (85.1)	590 (86.6)	97 (77.0)	
R1	120 (14.9)	91 (13.4)	29 (23.0)	
T Stage, %				**<0.01**
T1	348 (43.1)	334 (49.0)	14 (11.1)	
T2	241 (29.9)	201 (29.5)	40 (31.7)	
T3	154 (19.1)	106 (15.6)	48 (38.1)	
Positive lymph nodes (%)	177 (21.9)	118 (17.3)	59 (46.8)	**<0.01**
No. of lymph nodes retrieved (IQR)	9 (4–15)	9 (4–15)	10 (5–16)	0.29

BMI, body mass index; ASA, American Society of Anesthesiologists Classification; CD, Clavien-Dindo; LVI, lymph node invasion; PNI, perineural invasion. Shown in bold are the variables with a statistically significant difference between patients with and without recurrences (*p* < 0.05).

**Table 2:** Patient and tumor characteristics of patients with early versus late recurrence.

Characteristics	Early Recurrence (*n* = 49)	Late Recurrence (*n* = 77)	*p*
Patient			
Male, %	21 (42.9)	41 (53.2)	0.26
Age, ± SD	58 (49–69)	58 (47–66)	0.51
BMI, ± SD	26 (23–30)	24 (22–28)	0.28
ASA, %			0.73
I	3 (6.1)	9 (11.7)	
II	21 (42.9)	29 (37.7)	
III	22 (44.9)	35 (45.5)	
IV	1 (2.0)	1 (1.3)	
Tumor			
Functional tumor, %	4 (8.2)	5 (6.5)	0.70
Symptomatic, %	24 (49.0)	35 (45.5)	0.31
Tumor size, IQR, cm	4.7 (2.7–7.5)	3.5 (2.5–6.0)	0.20
Tumor Location, %			0.74
Head	23 (46.9)	34 (44.2)	
Body	9 (18.4)	19 (24.7)	
Tail	16 (32.7)	24 (31.2)	
Multiple tumors, %	0	0	-
Type of Resection			0.11
Pancreatoduodenectomy	21 (42.9)	37 (48.1)	
Distal pancreatectomy	21 (42.9)	33 (42.9)	
Enucleation/Central	6 (12.2)	2 (2.6)	
Major venous/arterial resection	6 (12.2)	7 (9.1)	0.87
Complications CD grade ≥ 3	16 (32.7)	22 (28.6)	0.95
Pathological			
Tumor Grade, %			0.92
G1	14 (28.6)	24 (31.2)	
G2	19 (38.8)	34 (44.2)	
LVI, %	18 (36.7)	26 (33.8)	0.74
PNI, %	10 (20.4)	16 (20.8)	0.47
Resection Margin, %			0.58
R0	39 (79.6)	58 (75.3)	
R1	10 (20.4)	19 (24.7)	
T Stage, %			0.28
T1	6 (12.2)	8 (10.4)	
T2	12 (24.5)	28 (36.4)	
T3	24 (49.0)	24 (31.2)	
Positive lymph nodes (%)	23 (46.9)	36 (46.8)	0.78
No. of lymph nodes retrieved (IQR)	11 (5–17)	8 (4–16)	0.27

BMI, body mass index; ASA, American Society of Anesthesiologists Classification; CD, Clavien-Dindo; LVI, lymph node invasion; PNI, perineural invasion.

**Table 3:** Risk factors for recurrence in patients with a pNET.

	**Bivariate**		**Multivariate**	
**Recurrence**	**Odds Ratio (95% CI)**	***p***	**Odds Ratio (95% CI)**	***p***
Age, >65 vs. ≤65	1.05 (0.69–1.60)	0.80		
Male	1.05 (0.72–1.54)	0.79		
Symptomatic	1.53 (0.98–2.39)	**0.06**	1.15(0.58–2.30)	0.68
Functional status	0.56 (0.28–1.15)	0.11		
Tumor size (cm)	1.23 (1.16–1.30)	**<0.01**	1.17 (1.05–1.30)	**0.004**
Margin status: R0 vs. R1	1.94 (1.21–3.10)	**0.006**	0.89 (0.38–2.09)	0.91
Complications CD ≥3	1.50 (0.91–2.47)	0.12		
Tumor grade, G1 vs. G2	3.52 (2.23–5.55)	**<0.01**	2.82 (1.38–5.79)	**0.005**
LVI	5.00 (3.00–8.32)	**<0.01**	1.52 (0.67–3.47)	0.32
PNI	2.63 (1.53–4.51)	**<0.01**	0.96 (0.44–2.08)	0.91
Positive lymph nodes	3.68 (2.45–5.54)	**<0.01**	2.32 (1.02–5.25)	**0.045**
	**Bivariate**		**Multivariate**	
**Early Recurrence (≤18 months)**	**Odds Ratio (95% CI)**	***p***	**Odds Ratio (95% CI)**	***p***
Age, >65 vs. ≤65	1.36 (0.74–2.51)	0.32		
Male	1.26 (0.71–2.25)	0.44		
Symptomatic	1.21 (0.65–2.25)	0.57		
Functional status	0.70 (0.24–1.99)	0.50		
Tumor size (cm)	1.23 (1.15–1.32)	**<0.01**	1.20 (1.05–1.37)	**0.007**
Margin status: R0 vs. R1	1.51 (0.73–3.11)	2.64		
Complications CD ≥ 3	1.52 (0.32–3.19)	0.27		
Tumor grade, G1 vs. G2	3.42 (1.66–7.01)	0.001	2.78 (0.96–8.07)	0.06
LVI	4.2 (2.01–8.65)	<0.01	0.97 (0.30–3.16)	0.96
PNI	2.14 (0.99–4.62)	0.05	0.60 (0.20–1.83)	0.37
Positive lymph nodes	3.96 (2.16–7.28)	<0.01	4.69 (1.41–15.58)	**0.01**
	**Bivariate**		**Multivariate**	
**Late Recurrence (>18 months)**	**Odds Ratio (95% CI)**	***p***	**Odds Ratio (95% CI)**	***p***
Age, >65 vs. ≤65	0.87 (0.51–1.49)	0.61		
Male	0.93 (0.58–1.49)	0.76		
Symptomatic	1.79 (0.98–2.26)	**0.06**	1.26 (0.53–3.00)	0.60
Functional status	0.52 (0.20–1.31)	0.17		
Tumor size (cm)	1.13 (1.06–1.21)	**<0.01**	1.08 (0.94–1.24)	0.26
Margin status: R0 vs. R1	2.04 (1.17–3.57)	**0.01**	1.46 (0.55–3.91)	0.45
Complications CD ≥ 3	1.40 (0.74–2.62)	0.30		
Tumor grade, G1 vs. G2	3.06 (1.77–5.29)	**<0.01**	2.55 (1.03–6.34)	**0.04**
LVI	4.61 (2.40–8.88)	**<0.01**	2.00 (0.71–5.95)	0.19
PNI	2.72 (1.36–5.45)	**0.005**	1.24 (0.48–3.19)	0.66
Positive lymph nodes	2.75 (1.68–4.48)	**<0.01**	1.10 (0.40–3.08)	0.85

Shown in bold are the variables selected for univariable (*p* < 0.1) and multivariable (*p* < 0.05) analysis; pNET, pancreatic neuroendocrine tumors. CD, Clavien-Dindo; LVI, lymph node invasion; PNI, perineural invasion; CI indicates confidence interval.

## Data Availability

Data can be made available upon request.

## Supplementary Materials

The following are available online at https://www.mdpi.com/article/10.3390/cancers13092242/s1, Table S1: Risk factors for pNETs > 2 cm.
