# Supplementary material for: Identifying Risk Factors and Patterns for Early Recurrence of Pancreatic Neuroendocrine Tumors: A Multi-Institutional Study
Source: Cancers (Basel). 2021 May 7;13(9):2242. doi: 10.3390/cancers13092242 (PMC8124896; doi:10.3390/cancers13092242)
Supplement: Supplementary file 1 [file cancers-13-02242-s001.zip › cancers-1206717-supplementary.pdf]

cancers

MDPI

Supplementary Materials: Identifying Risk Factors and Patterns for Early Recurrence of Pancreatic Neuroendocrine Tumors: A Multi-Institutional Study

Charlotte M. Heidsma [1,2], Diamantis I. Tsilimigras [1], Flavio Rocha [3], Daniel E. Abbott [4], Ryan Fields [5], George A. Poultsides [6], Clifford S. Cho [7], Alexandra G. Lopez-Aguiar [8], Zaheer Kanji [3], Alexander V. Fisher [4], Bradley A. Krasnick [5], Kamran Idrees [9], Eleftherios Makris [6], Megan Beems [7], Casper H. J. van Eijck [10], Elisabeth J. M. Nieveen van Dijkum [2], Shishir K. Maithel [8] and Timothy M. Pawlik [1,*]

Table S1. Risk factors for pNETs > 2 cm.

Early Recurrence (≤18 Months)	Bivariate Odds Ratio (95% CI)	*p*	Multivariate Odds Ratio (95% CI)	*p*
Age, >65 vs. ≤ 65	1.23 (0.61–2.79)	0.56		
Male	1.34 (0.70–2.58)	0.38		
Symptomatic	0.96 (0.48–1.94)	0.91		
Functional status	0.92 (0.27–3.16)	0.89		
Tumor size (cm)	1.18 (1.09–1.28)	**<0.01**	1.17 (1.01–1.36)	**0.034**
Margin status: R0 vs. R1	0.92 (0.39–2.15)	0.84		
Complications CD ≥3	1.05 (0.56–2.42)	0.91		
Tumor grade, G1 vs. G2	3.82 (1.56–9.35)	**0.003**	3.34 (1.02–11.00)	**0.047**
LVI	2.66 (1.15–6.18)	**0.03**	0.66 (0.20–2.14)	0.49
PNI	1.91 (0.82–4.45)	0.13		
Positive lymph nodes	2.60 (1.33–5.08)	**0.005**	4.84 (1.36–17.26)	**0.015**

Shown in bold are the variables selected for multivariate ($p < 0.1$) analysis.
